# First record of the genus *Oodera* Westwood, 1874 (Hymenoptera, Pteromalidae, Cleonyminae, Ooderini) from the Arabian Peninsula, with the description of four new species

**DOI:** 10.3897/zookeys.874.35935

**Published:** 2019-09-03

**Authors:** Ahmed M. Soliman, Neveen S. Gadallah, Hathal M. Al Dhafer

**Affiliations:** 1 Plant Protection Department, College of Food and Agriculture Sciences, King Saud University, PO BOX 2460, Riyadh 11451, Saudi Arabia King Saud University Riyadh Saudi Arabia; 2 Zoology Department, Faculty of Science (Boys), Al-Azhar University, PO Box 11884, Nasr City, Cairo, Egypt Al-Azhar University Cairo Egypt; 3 Entomology Department, Faculty of Science, Cairo University, Giza, Egypt Cairo University Giza Egypt

**Keywords:** *
Acacia
*, Oman, parasitic wasp, Saudi Arabia, systematics, xylophagous hosts

## Abstract

The genus *Oodera* Westwood, 1874 (Hymenoptera, Pteromalidae, Cleonyminae) is recorded for the first time for the Arabian Peninsula, from the Kingdom of Saudi Arabia and the Sultanate of Oman. The present study is based on specimens reared from xylophagous beetle larvae of the family Buprestidae (Coleoptera) infesting dead *Acacia* trees from Al-Dakhiliyah and Dhofar governorates in Oman and Al-Baha, Asir and Riyadh regions in Saudi Arabia. Four new species, *Oodera arabica***sp. nov.**, *O. omanensis***sp. nov.**, *O. rapuzzii***sp. nov.**, and *O. similis***sp. nov.** are described, illustrated and compared with closely related *Oodera* species. An illustrated key and the xylophagous host records of the species are also provided.

## Introduction

*Oodera* Westwood, 1874 (Hymenoptera, Pteromalidae, Cleonyminae) is a relatively small genus comprising currently twenty valid species (Werner and Peters 2018). It includes different-sized species (3.6−17 mm) (Werner and Peters 2018). Based on Holt et al. (2013), they are distributed in the Oriental region (eight species), Afrotropical region (six species), Palaearctic region (six species) and Nearctic region (one species) (Noyes 2018; Werner and Peters 2018). However, the relatively recent report of *O. formosa* (Giraud) from the United States of America results from an accidental introduction from Europe (Werner and Peters 2018). Almost all *Oodera* species are reported as parasitoids of xylophagous beetle larvae of the families Buprestidae and Curculionidae (Coleoptera, Scolytinae) (Bouček 1958; Bouček and Rasplus 1991; Yang 1996; Gibson 2003; Werner and Peters 2018). Other details about their biology are still unknown (Werner and Peters 2018).

The phylogenetic status of *Oodera* has remained in dispute for a long time (Gibson 1989). It has been proposed as forming a link or a bridge between Cleonyminae (Pteromalidae) and Eupelminae (Eupelmidae) (Bouček 1958, 1988; Graham 1969) and has been classified and keyed in Eupelmidae rather than Cleonyminae by some authors (Ashmead 1904; Nikol’skaya 1952; Graham 1969). It was transferred from Eupelmidae to Pteromalidae by Bouček (1958) who established the monotypic tribe Ooderini for the genus in the subfamily Cleonyminae, family Pteromalidae (Heraty et al. 2013). The presence of a peculiar system of spines and spine-like setae along the ventral margin of the profemur, and the absence of a flexible transscutal articulation both support the monophyly of *Oodera* (Gibson 2003). On the other hand, the very distinctive structures and modifications of its middle legs (thickened mesotibial spur; presence of mesotarsal pegs; the presence of a membranous area anterior to each mesocoxa) might support the hypothesis of it being a sister of Eupelmidae or some part of it (Gibson 1989).

The first comprehensive work dealing with the taxonomy of the genus *Oodera* was that of Werner and Peters (2018), who revised the world species based on the morphological examination of 115 specimens. They reported twenty valid species for the genus, of which ten species were described as new to science. An illustrated key to all species, re-description of the other valid ones, with taxonomic treatments to some of them, were also given.

In the present study, *Oodera* is recorded for the first time for the fauna of the Arabian Peninsula, from Oman and Saudi Arabia. Specimens were reared from the dead wood of *Acacia* sp. trees. Four new species are described and illustrated. An illustrated key and the xylophagous hosts of species are also provided.

## Material and methods

The present study is based on 25 specimens reared from dead wood of *Acacia* sp. trees collected from Al-Dakhiliyah and Dhofar governorates in Oman and Al-Baha, Asir and Riyadh regions in Saudi Arabia, as follows:

Oman, Al-Dakhiliyah: Al-Hamra (23°10'26"N, 57°08'49"E, alt. 825 m).

Oman, Dhofar: Mirbat (17°11'09"N, 54°59'31"E, alt. 500 m); Rawiyya (17°20'45"N, 54°03'57"E , alt. 650 m).

Saudi Arabia, Al-Baha: Shada Al-Ala Natural Reserve (19°51'40"N, 41°18'16"E, alt. 1248 m); The Ain Village (19°55'47"N, 41°26'38"E, alt. 760 m); Wadi Tourabah (20°11'36"N, 41°17'50"E, alt. 1830 m); Wadi Shoqab (20°40'27"N, 41°15'02"E, alt. 1440 m); Wadi Yabah (19°16'32"N, 41°48'33"E, alt. 440 m).

Saudi Arabia, Asir: Wadi Sabian (28 km S. Muhayil) (18°17'55"N, 42°07'41"E, alt. 809 m).

Saudi Arabia, Riyadh: Wadi Al Hesiyah (40 km NW Riyadh) (24°55'22"N, 46°12'15"E, alt. 790 m).

The collected specimens were pinned directly for further study. Identification of the new species was made with the help of Werner and Peters’ key (2018). Abbreviations used for measurements are based on Werner and Peters (2018), as follows: **bdy.l** = body length; **cor.l** = corona length; **cor.w** = corona width; **F1, F2, F3** = first, second, third flagellomeres; **hea.h** = head height (frontal view); **hea.l** = maximum length of head (lateral view); **hea.w** = maximum width of head (frontal view); **eye.h** = height of eye (lateral view); **msp.l** = malar space; **eye.d** = shortest distance between eyes; **POL** = shortest distance between posterior ocelli (dorsal view); **OOL** = shortest distance between posterior ocellus and eye (dorsal view); **no.l** = pronotum length; **no.w** = pronotum maximum width; **msc.l** = mesoscutum length; **msc.w** = mesoscutum maximum width (= mesonotum width); **msn.l** = mesonotum length; **sct.l** = mesoscutellum length; **sct.w** = mesoscutellum width; **ppd.l** = propodeal length; **fm1.l** = profemur length; **fm1.w** = profemur width; **mav.l** = marginal vein length; **pmv.l** = postmarginal vein length; **mts.l** = metasomal length; **mts.w** = metasomal width; **ovp.l** = ovipositor length. Description format, characters definition, and ranges of measured ratios follow Werner and Peters (2018) to facilitate comparison. Body-sculpture terminology follows Harris (1979).

Photographic images were taken using a Canon EOS 70D camera attached to a Leica MZ 125 stereomicroscope. Individual source images were then stacked using HeliconFocus v6.22 (HeliconSoft Ltd) extended depth of field software. Further image processing was done using the software Adobe Photoshop CS5.1 (ver. 12.1x 32) and Adobe Photoshop Lightroom 5.2. Morphological measurements of the different parts were made with the help of a Zeiss Stemi 2000-C stereomicroscope with an ocular micrometer (100 lines per mm). Body part measurements were taken with the same magnification (20× eyepiece, 2.5× objective) for calculating different body ratios accurately and facilitate comparison. The detailed description for each species under study is based on the holotype specimen; for the diagnosis, all specimens under study were measured, and the minimum and maximum values are used.

**Figure 1.**
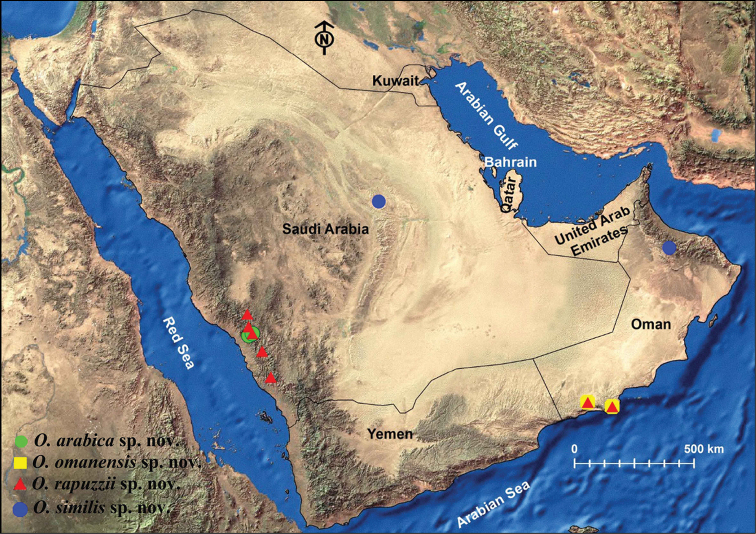
Distributional map of collection localities of *Oodera* species in the Arabian Peninsula.

The distribution of the prospected sites is plotted using ArcGIS 10.4. (Fig. 1). The type specimens of the new species are deposited in King Saud University Museum of Arthropods (**KSMA**), Plant Protection Department, College of Food and Agriculture Sciences, King Saud University, Riyadh, Saudi Arabia.

## Systematic account

### Oodera

Taxon classificationAnimaliaHymenopteraPteromalidae

Westwood, 1874

EC27E66604035317BF48323B0E803B5D

Oodera Westwood, 1874. Thesaurus Entomologicus Oxoniensis: 145. Type species: Oodera gracilis Westwood; subsequent designation by Ashmead (1904: 288).
Stellophora Risbec, 1951. Mem. Inst. Er. Afr. Noire 63: 239. Type species: Stellophora magnifica Risbec by monotypy. Synonymized by Bouček (1958: 375).

#### Diagnosis.

Individuals of *Oodera* are diagnosed by the following combination of character states: head with deep scrobes in the form of an inverted V; parascrobal area of the head crested (= corona of Werner and Peters 2018); mesosoma dorsally flattened, with pronotum (no.) usually longer than wide, pentagonal (rounded in few cases), without differentiated collar, widened anteriorly and narrowed towards mesoscutum; mesoscutum (msc.) with star-like grooves, arranged radially from almost one point, notauli sulcate, V-shaped, extended to anterior margin of mesoscutellum; axillae conspicuously large, triangular, greatly advanced anterior to mesoscutellum; mesoscutellum (sct.) longitudinally ridged dorsally, with smoother coraceous apex; profemur (fm1.) distinctly enlarged, oval-shaped, with a row of oblique strong black bristles and a comb of peculiar pegs along its outer ventral margin; protibia (tb1.) curved, strongly carinate along its dorsal and ventral margins; mesocoxae with small membranous area anterior to each one; postmarginal vein (pmv.) of forewing slightly longer or slightly shorter than marginal vein (mav.); metasomal petiole very short, membranous medio-ventrally; ovipositor (ovp.) sheaths varying in length among the different species, from shorter than, to distinctly longer than metasomal length (mts.l) (Bouček 1958, 1988; Gibson 1989, 2003; Bouček and Rasplus 1991; Werner and Peters 2018).

### Key to *Oodera* species of the Arabian Peninsula (male of *O. arabica* is unknown)

**Table:** 

1	Body medium-sized (7.0−7.2 mm); antenna with scape, pedicel and basal half of F1 red, rest of flagellum black (Fig. 3A); pronotum 1.20−1.25× as wide as long, with broadest part at midlength (Figs 8A, 9A); mesoscutellum dull, black, with faint purple tint, and entirely lineate (median lines straight) (Figs 8A, 9E); apical segment of maxillary palp relatively long, angled baso-ventrally, lined ventrally with dense short whitish spines together with scattered long setae (Fig. 7A); metasoma 1.92× as long as wide (Fig. 2A); stigmal vein slender, straight, with stigma slightly roundly swollen apically (Fig. 10A)	***Oodera arabica* sp. nov.**
–	Body small-sized (4.5−6.5 mm); antenna with only scape or part of it red, rest of antenna black (Fig. 3B−D); pronotum as wide as long, with broadest part before or behind midlength (Figs 8B−D, 9B−D); mesoscutellum shiny, metallic green or coppery, and lineate on anterior three-fourths or slightly more, but at least finely areolate before frenal line (median lines converging) (Figs 8B−D, 9F−H); apical segment of maxillary palp distinctly shorter, smoothly rounded baso-ventrally, without such short spines along its ventral margin (Fig. 7B−D); metasoma 2.12−2.45× as long as wide (Fig. 2B−D); stigmal vein relatively thick, curved, with smoothly quadrate stigma (Fig. 10B−D)	**2**
2	Scape with basal two-thirds red and apical third black (Fig. 5C); horizontal crests of corona distinctly prominent (high) and widely spaced (Figs 5G, 6C); pronotum with anterior margin rounded (Figs 8C, 9C); mesoscutellum red-violet anteriorly (Figs 8C, 9G), normal to slender (0.65−0.75× as long as wide), lineate on anterior two-thirds and finely areolate on posterior third, and with posterior margin narrowly rounded (Figs 8C, 9G); propodeum with a smooth ridge in front of setose area (Figs 8C, 9G); body robust to slender (mesonotum 1.33−1.45× as long as wide)	***Oodera rapuzzii* sp. nov.**
–	Scape of antenna entirely red (Fig. 3D) to red-brown (Fig. 5B) or sometimes with black tint apically; horizontal crests of corona less prominent (low) and narrowly spaced (Figs 5F, H, 6B, D); pronotum with anterior margin truncated or nearly so (Fig. 9B, D); mesoscutellum bright metallic green to blue-green anteriorly (Fig. 9F, H), normal (0.60−0.72× as long as wide), almost completely lineate though finely areolate slightly before frenal line, and with posterior margin broadly rounded (Fig. 9F, H); propodeum laterally with costate ridge in front of setose area (Fig. 9F, H); body robust (mesonotum 1.28−1.34× as long as wide)	**3**
3	Forewing partly infumate (Fig. 10B); pronotum narrow anteriorly, with broadest part behind midlength (Fig. 9B); face bluish to purplish (Fig. 5B, F); mesoscutellum with posterior half purplish (Fig. 9F); propodeum medium (ppd.l/msc.l 0.13−0.15); ovipositor rather long (16−17× as long as metasoma length); volsella of male genitalia with four teeth (Fig. 11D)	***Oodera omanensis* sp. nov.**
–	Forewing hyaline (Fig. 10D); pronotum broad anteriorly, with broadest part slightly before midlength (Fig. 9D); face green with coppery tint (Fig. 5D, H); mesoscutellum entirely green (Fig. 9H); propodeum large (pd.l/msc.l 0.17−0.21); ovipositor short (0.13−0.14× as long as metasoma length); volsella of male genitalia with five teeth (Fig. 11F)	***Oodera similis* sp. nov.**

### Oodera arabica

Taxon classificationAnimaliaHymenopteraPteromalidae

Gadallah & Soliman sp. nov.

7A967BA3A2D056DFAC689BBED849A1E3

http://zoobank.org/6D4D5A30-8081-4B52-988B-6AEC6BB41156

Figs 2A , 3A , 4A , 5 (A, E), 6A, 7A, 8A, 9(A, E), 10A

#### Material examined.

**Holotype** ♀: SAUDI ARABIA, Al-Baha (Al-Mikhwah, Shada Al-Ala Natural Reserve), 29.iii.2017, leg. D. Baiocchi, e.l. *Acacia* [KSMA]. **Paratype** 1♀: SAUDI ARABIA, Al-Baha (Al-Mikhwah, The Ain Village), 13.iv.2016, leg. D. Baiocchi, e.l. *Acacia* sp. [KSMA].

**Figure 2.**
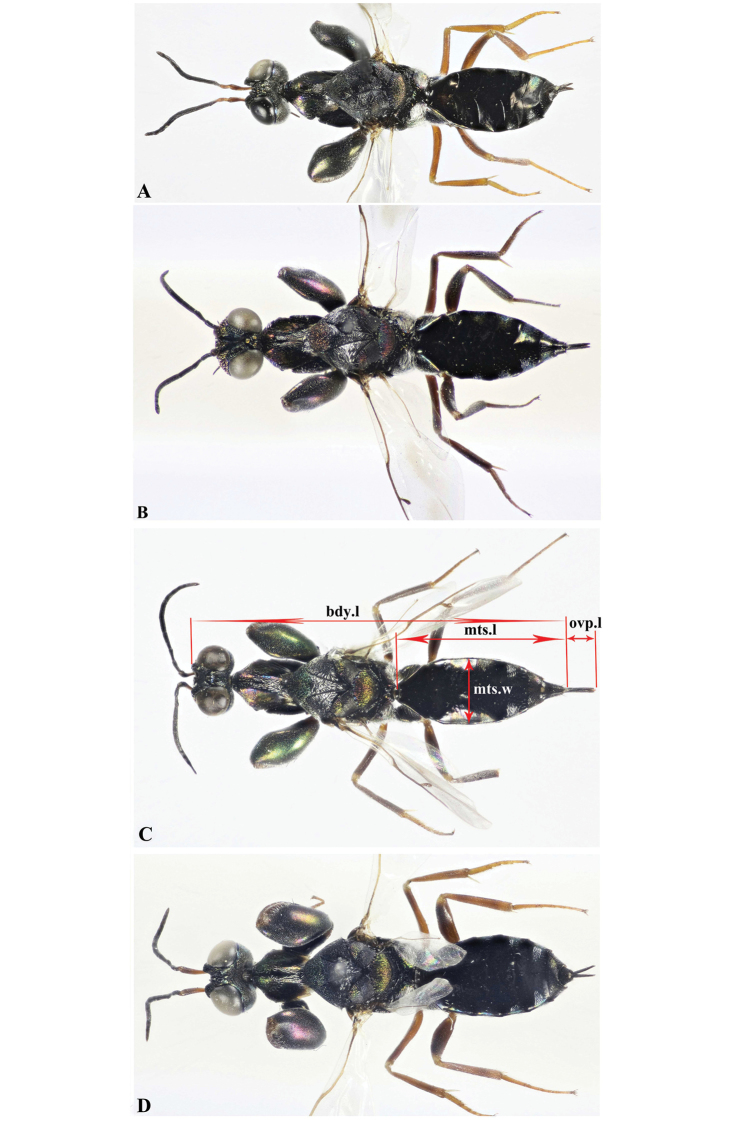
Dorsal habitus, Holotype **A***Oodera arabica* sp. nov. **B***Oodera omanensis* sp. nov. **C***Oodera rapuzzii* sp. nov. **D***Oodera similis* sp. nov.

**Figure 3.**
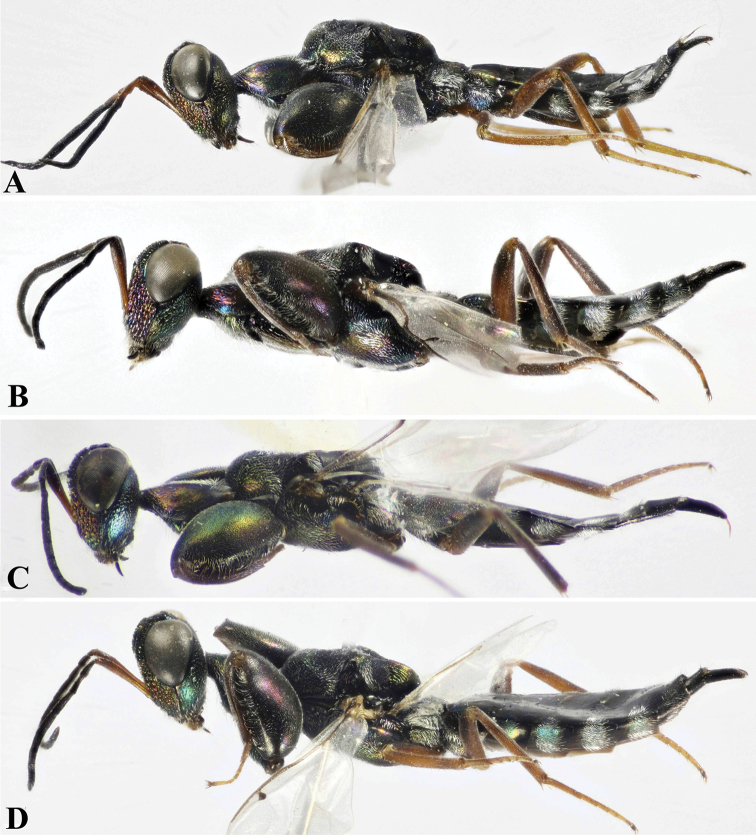
Lateral habitus, Holotype **A***Oodera arabica* sp. nov. **B***Oodera omanensis* sp. nov. **C***Oodera rapuzzii* sp. nov. **D***Oodera similis* sp. nov.

**Figure 4.**
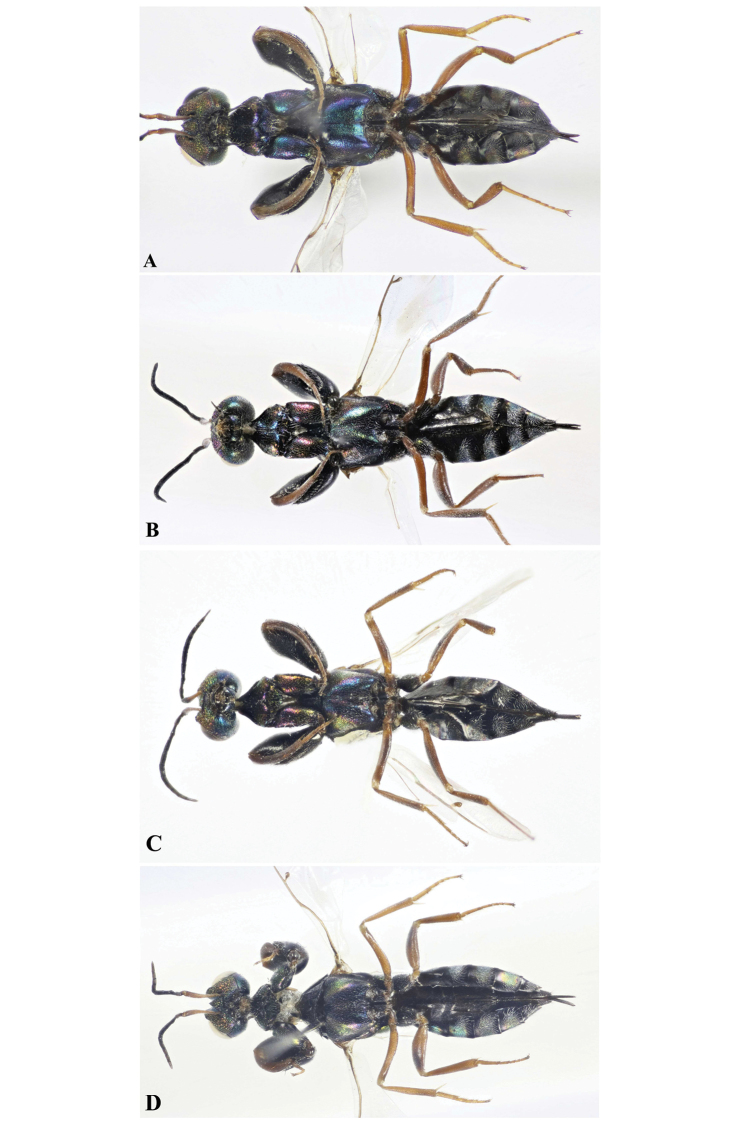
Ventral habitus, Holotype **A***Oodera arabica* sp. nov. **B***Oodera omanensis* sp. nov. **C***Oodera rapuzzii* sp. nov. **D***Oodera similis* sp. nov.

#### Diagnosis

(female) (*N* = 2). See Table 1.

**Table 1.** Diagnosis of *Oodera* species in the Arabian Peninsula.

Character	*O. arabica* sp. nov. (*N* = 2)	*O. omanensis* sp. nov. (*N* = 4)	*O. rapuzzii* sp. nov. (*N* = 16)	*O. similis* sp. nov. (*N* = 3)
Bdy.l (in mm)	Medium-sized (7.0−7.2)	Small-sized (5.5−6.3)	Small-sized (4.5−5.6)	Small-sized (6.3−6.5)
Body shape (msn.l/msc.w)	Robust (1.25−1.29) (Fig. 8A)	Robust (1.3−1.34) (Fig. 8B)	Robust to slender (1.33−1.45) (Fig. 8C)	Robust (1.28−1.30) (Fig. 8D)
Head shape in lateral view (hea.h/hea.l)	Rounded (1.34−1.38)	Rounded (1.40−1.44)	Rounded (1.35−1.36)	Rounded (1.37−1.38)
Eye size (eye.h/hea.h)	Large (0.72−0.74)	Large (0.70−0.75)	Large (0.70−0.72)	Large (about 0.75)
Corona shape (cor.l/cor.w), its structure	Thick (3.08−3.60), with interrupted structure (Fig. 6A)	Thick (3.33−3.50), with interrupted structure (Fig. 6B)	Thick (3.25−4.0), with interrupted structure (Fig. 6C)	Thick (3.11−3.40), with interrupted structure (Fig. 6D)
Length and shape of apical segment of maxillary palp	Relatively long, distinctly angulate baso-ventrally, lined ventrally with dense short whitish spines together with scattered long setae (Fig. 7A)	Relatively short, evenly rounded (not angulate) baso-ventrally, without such short spines along its ventral margin (Fig. 7B)	Relatively short, evenly rounded (not angulate) baso-ventrally, without such short spines along its ventral margin (Fig. 7C)	Relatively short, evenly rounded (not angulate) baso-ventrally, without such short spines along its ventral margin (Fig. 7D)
Pronotum length (according to its width), its anterior margin	Wider than long (1.20−1.25×), with anterior margin truncate (Fig. 9A)	As long as wide, with anterior margin truncate (Fig. 9B)	As long as wide, anterior margin rounded (Fig. 9C)	As long as wide, anterior margin truncate (Fig. 9D)
Pronotum broadest part	At midlength (Fig. 9A)	Behind midlength (Fig. 9B)	Slightly behind midlength (Fig. 9C)	Slightly before midlength (Fig. 9D)
Mesoscutellum shape (sct.l/sct.w), its anterior margin	Normal (0.60−0.62), anterior margin hardly convex (Fig. 9E)	Normal (0.62−0.64), anterior margin hardly convex (Fig. 9F)	Normal to slender (0.65− 0.75), anterior margin hardly convex (Fig. 9G)	Normal (0.60−0.72), anterior margin hardly convex (Fig. 9H)
Mesoscutellum sculpture	Entirely lineate (median lines straight) (Fig. 9E)	Almost completely lineate, finely areolate slightly before frenal line (median lines converging) (Fig. 9F)	Lineate on anterior two-thirds and finely areolate on posterior third (median lines converging) (Fig. 9G)	Almost completely lineate, finely areolate slightly before frenal line (median lines converging) (Fig. 9H)
Propodeum size ppd.l/msc.l	Medium (about 0.15) (Fig. 8A)	Medium (0.13−0.15) (Fig. 8B)	Medium (0.13−0.14) (Fig. 8C)	Large (0.17−0.21) (Fig. 8D)
Profemur shape (fm1.l/fm1.w)	Robust to medium (1.90−2.08)	Robust to medium (1.92−2.00)	Robust (1.95−2.00)	Robust (1.95−2.00)
Forewing	Hyaline (Fig. 10A)	Partly slightly infumate (Fig. 10B)	Hyaline (Fig. 10C)	Hyaline (Fig. 10D)
Marginal vein length mav.l/pmv.l	Short to medium (0.87−0.92) (Fig. 10A)	Medium (0.92−1.00) (Fig. 10B)	Medium (0.95−1.00) (Fig. 10C)	Medium (0.95−1.00) (Fig. 10D)
Metasoma length mts.l/ bdy.l	Short (0.42−0.43) (Fig. 2A)	Short (about 0.43) (Fig. 2B)	Short to medium (0.44−0.47) (Fig. 2C)	Short (0.42−0.45) (Fig. 3D)
Ovipositor length ovp.l/ mts.l	Short (1.00−0.13) (Fig. 2A).	Rather long (0.16−0.17) (Fig. 2B)	Rather long (0.16−0.19) (Fig. 2C)	Short (0.13−0.14) (Fig. 3D)
Volsella teeth (male genitalia)	unknown	Four (Fig. 11D)	Four (Fig. 11E)	Five (Fig. 11F)

#### Description.

Female (holotype): Body length 7.2 mm (excluding the ovipositor).

**Figure 5.**
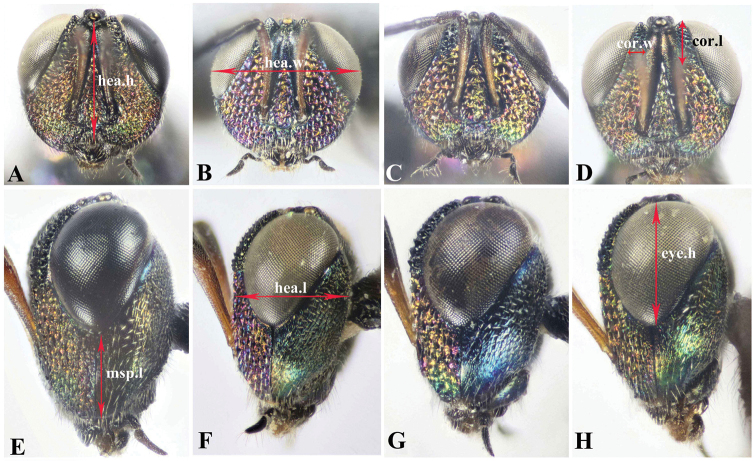
Frontal and lateral view of head, Holotype **A,E***Oodera arabica* sp. nov. **B,F***Oodera omanensis* sp. nov. **C,G***Oodera rapuzzii* sp. nov. **D,H***Oodera similis* sp. nov.

**Figure 6.**
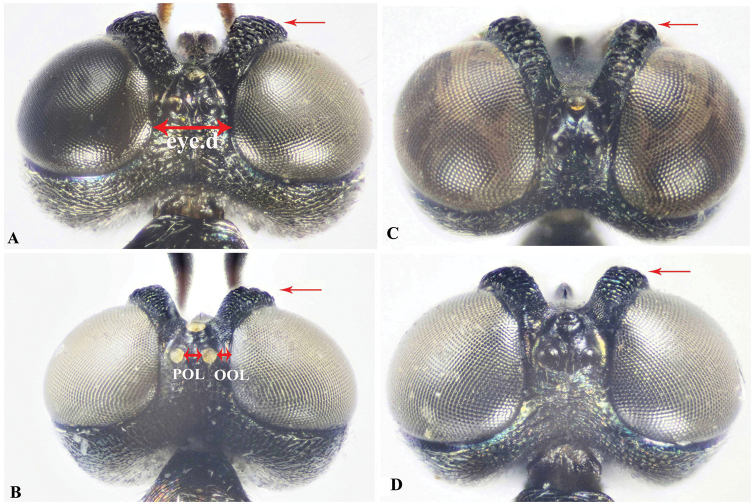
Dorsal view of head, Holotype **A***Oodera arabica* sp. nov. **B***Oodera omanensis* sp. nov. **C***Oodera rapuzzii* sp. nov. **D***Oodera similis* sp. nov. (coronal structure indicated).

**Figure 7.**
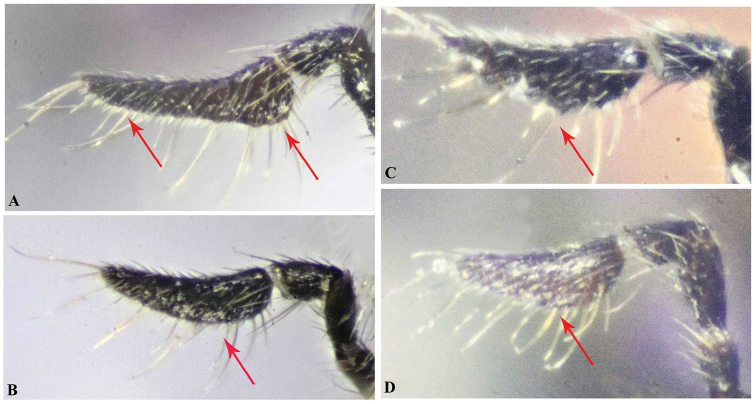
Maxillary palp, Holotype **A***Oodera arabica* sp. nov. **B***Oodera omanensis* sp. nov. **C***Oodera rapuzzii* sp. nov. **D***Oodera similis* sp. nov. (apical segment of maxillary palp indicated).

**Figure 8.**
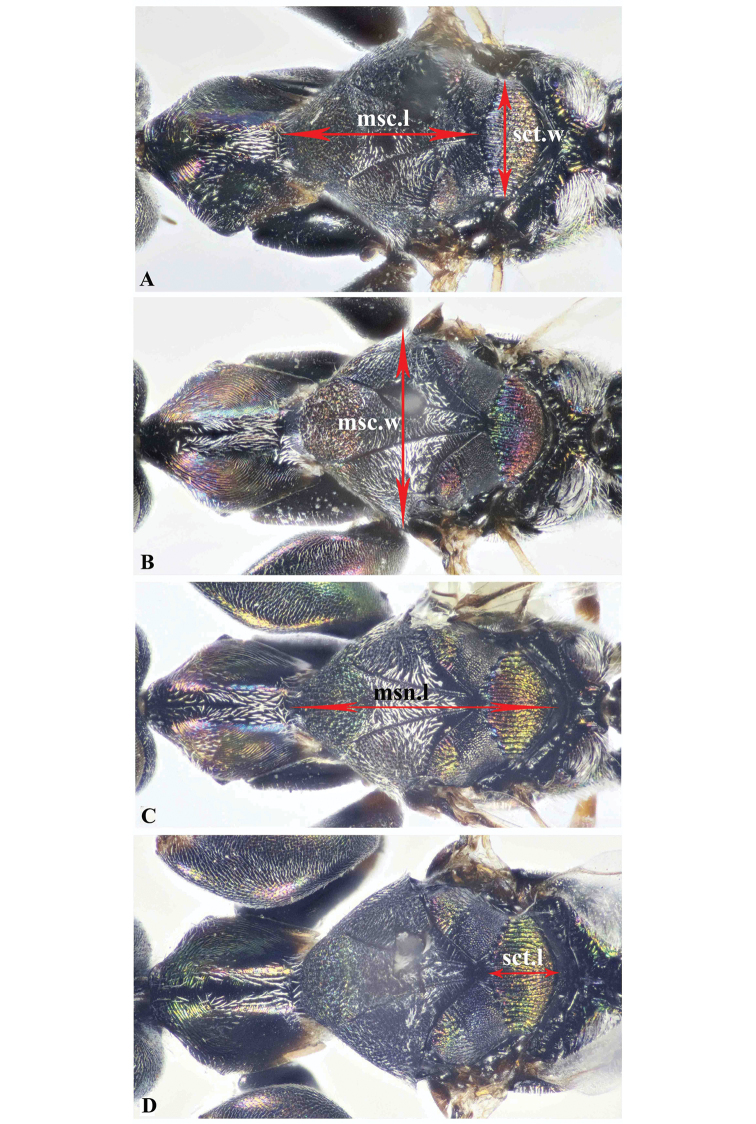
Dorsal aspect of mesosoma, Holotype **A***Oodera arabica* sp. nov. **B***Oodera omanensis* sp. nov. **C***Oodera rapuzzii* sp. nov. **D***Oodera similis* sp. nov.

**Figure 9.**
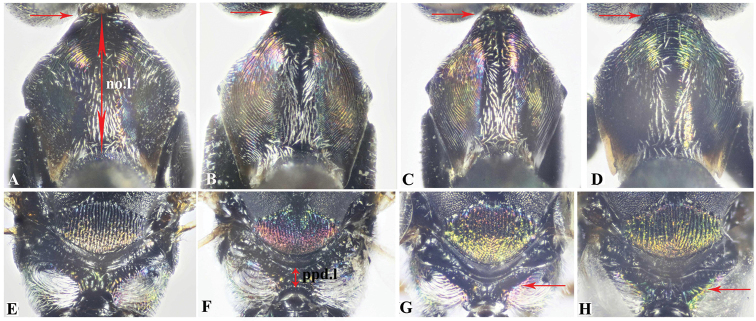
**A−D** Dorsal aspect of pronotum, Holotype **A***Oodera arabica* sp. nov. **B***Oodera omanensis* sp. nov. **C***Oodera rapuzzii* sp. nov. **D***Oodera similis* sp. nov. **E−H** Mesoscutellum, metanotum and propodeum **E***Oodera arabica* sp. nov. **F***Oodera omanensis* sp. nov. **G***Oodera rapuzzii* sp. nov. **H***Oodera similis* sp. nov. (anterior margin of pronotum indicated in **A−D**, propodeal lateral ridge indicated in **G, H**).

**Figure 10.**
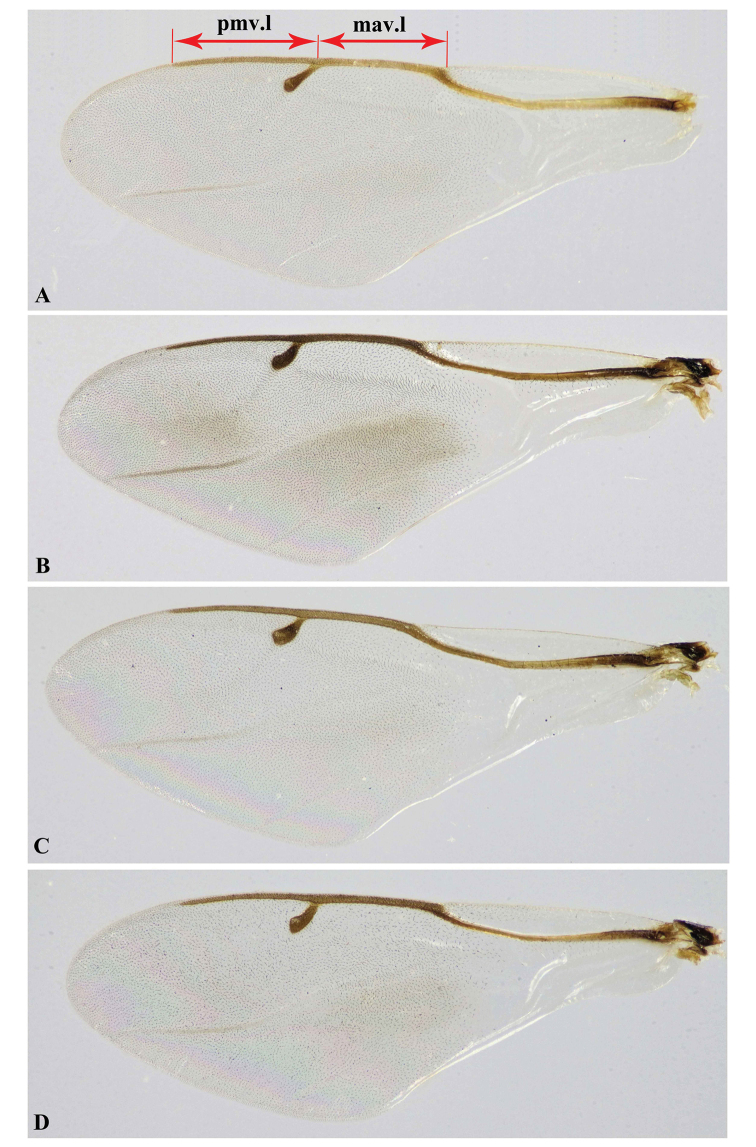
Forewing, Holotype **A***Oodera arabica* sp. nov. **B***Oodera omanensis* sp. nov. **C***Oodera rapuzzii* sp. nov. **D***Oodera similis* sp. nov.

***Colour.*** Head black with strong coppery and green luster on face and faint green tint on gena (Figs 5A, E, 6A); scape, pedicel and basal half of FI red, rest of antenna black (Fig. 3A); maxillary and labial palpi dark brown to black (Fig. 7A). Mesosomal dorsum black with extremely faint purple and blue-green luster on pronotum, anterior part of axilla, mesoscutellum and propodeum (Figs 8A, 9A, E); mesosomal venter and coxae blue, midcoxa blackish (Fig. 4A); protrochanter and profemur black, the latter with slight blue-green tint on outer side (Fig. 3A); meso- and metatrochanters, tibiae and tarsi red, tarsi lighter (Figs 3A, 4A). Metasoma black, Gt2−5 with patches of green and slight coppery luster laterally (Figs 2A, 3A, 4A). Wings hyaline, veins yellow to light brown (Fig. 10A).

***Head.*** 1.6× as wide as long, hea.w 4.5× eye.d (Fig. 5A); face setiferous foveate-reticulate, setae lanceolate, white and short (Fig. 5A); msp.l 0.37× head height (Fig. 5E); corona 0.6× as long as eye.h (Fig. 5A); POL 1.37× OOL (Fig. 6A); scape 3.23× as long as pedicel; clava 0.15× as long as funicle; flagellum 1.2× as long as hea.w; F1 0.6× as long as F2; F2 1.12× as long as F3.

***Mesosoma.*** Pronotum pentagonal, 0.48× as long as mesonotum (Fig. 8A); mesonotum 1.38× as long as mesoscutum (Fig. 8A); mesoscutum 0.9× as long as wide (Fig. 8A); mesoscutellum 0.38× as long as mesoscutum (Fig. 8A); profemur 1.4× as long as protibia.

***Forewing*** (Fig. 10A). Forewing 2.87× as long as wide; costal cell 0.35× as long as forewing; marginal vein 0.19× as long as forewing; marginal vein 3.36× as long as stigmal vein; postmarginal vein 3.86× as long as stigmal vein.

***Metasoma*** (Fig. 2A, 3A, 4A). mts.l./mts.w = 1.92.

#### Male.

Unknown.

#### Host record.

Anthaxia (Haplanthaxia) kneuckeri zabranskyi Bílý, 1995 (Buprestidae).

#### Distribution.

Saudi Arabia (Al-Baha region).

#### Remarks.

The new species resembles the Afrotropical species *O. mkomaziensis* Werner & Peters (Tanzania) and *O. namibiensis* Werner & Peters (Namibia) in having body medium-sized (7.0−7.2 mm in length), head and mesosoma uniformly black with tinges of dark green and coppery or purple, eye large (eye.h/hea.h 0.72−0.74), corona thick (cor.l/cor.w 3.08−3.60), with structure interrupted and propodeum medium (ppd.l/msc.l 0.15). It differs from *O. mkomaziensis* in the following: body robust, msn.l/msc.w 1.25−1.29 (in *mkomaziensis* slender, msn.l/msc.w 1.5); head rounded, 1.34−1.38× as high as long (oval, 1.56× as high as long in *mkomaziensis*); pronotum pentagonal (oval in *mkomaziensis*); mesoscutellum entirely lineate (in *mkomaziensis* lineate in anterior two-thirds, finely areolate in posterior third); metasoma short, 0.42−0.43× as long as body (in *mkomaziensis* longer, 0.52× as long as body). The new species also differs from *O. namibiensis* in the following: forewing hyaline (partly infumate in *namibiensis*); antennal scape and pedicel and basal half of F1 are red, rest of antenna black (scape yellow, darkened apically, rest of antenna black in *namibiensis*); metasoma short, 0.42−0.43× as long as body length (in *namibiensis* medium to long, 0.49−0.55× as long as body length).

#### Etymology.

Named in reference to the country of Saudi Arabia, where the type specimen was collected.

### Oodera omanensis

Taxon classificationAnimaliaHymenopteraPteromalidae

Soliman & Gadallah sp. nov.

CD11930FA246547285438EDE2F13BF9C

http://zoobank.org/3F7B9623-168C-40E3-BA69-F66E66830648

Figs 2B , 3B , 4B , 5 (B, F), 6B, 7B, 8B, 9(B, F), 10B, 11(A, D)

#### Material examined.

**Holotype** ♀: OMAN, Dhofar (Rawiyya), 16.i.2018, leg. D. Baiocchi, e.l. *Acacia* sp. [KSMA]; **Paratypes**: 2♀ & 1♂: OMAN, Dhofar (Mirbat), 15−18.i.2018, leg. D. Baiocchi, e.l. *Acacia* sp. [KSMA].

#### Diagnosis.

Both sexes (*N* = 4). See Table 1.

#### Description.

Female (holotype): Body length 6.3 mm (excluding the ovipositor).

***Colour.*** Face purple with bluish reflections (Fig. 5B), corona and scrobal depression black with slight bluish tint, gena and occiput dark green (Figs 5B, F, 6B); scape red-brown, with slight black tint on apical fourth, rest of antenna, mandible, maxillary and labial palpi dark brown to black (Figs 3B, 5B, 7B). Mesosomal dorsum black with purple luster on pronotum, anterior third of mesoscutal median lobe, anterior part of axilla and on propodeum (Figs 8B, 9B, F); mesoscutellum purple, with slight green and blue luster anteriorly (Fig. 9F); mesosomal venter black, with blue and green luster on prosternum and mesopleuron respectively (Fig. 4B); coxae black with strong purple luster on procoxa and green on metacoxa respectively (Fig. 4B); protrochanter black, meso- and metatrochanters, tibiae and tarsi red-brown (Figs 3B, 4B); profemur black with purple tint on outer side (Fig. 3B). Metasoma black, tergites with patches of blue laterally (Figs 2B, 3B, 4B). Forewing partly slightly infumate, veins dark brown (Fig. 10B).

***Head.*** 1.7× as wide as long, hea.w 4.5× eye.d (Fig. 5B); face setiferous foveate-reticulate, setae lanceolate, white and short (Fig. 5B); msp.l 0.43× head height (Fig. 5F); corona 0.5× as long as eye.h (Fig. 5B); POL 1.4× OOL (Fig. 6B); scape 3× as long as pedicel; clava 0.11× as long as funicle; flagellum 1.25× as long as hea.w; F1 0.8× as long as F2; F2 hardly longer than F3.

***Mesosoma.*** Pronotum pentagonal, 0.53× as long as mesonotum (Fig. 8B); mesonotum 1.4× as long as mesoscutum (Fig. 8B); mesoscutum 0.95× as long as wide (Fig. 8B); mesoscutellum 0.4× long as mesoscutum (Fig. 8B); propodeum with costate ridge in front of the setose area (Fig. 9F); profemur 1.4× as long as protibia.

***Forewing*** (Fig. 10B). Forewing with dense and long setae, 2.8× as long as wide; costal cell 0.37× as long as forewing; marginal vein 0.2× as long as forewing; marginal vein 4× as long as stigmal vein; postmarginal vein 4.3× as long as stigmal vein.

***Metasoma*** (Figs 2B, 3B, 4B). mts.l/mts.w = 2.16.

#### Male.

**Figure 11.**
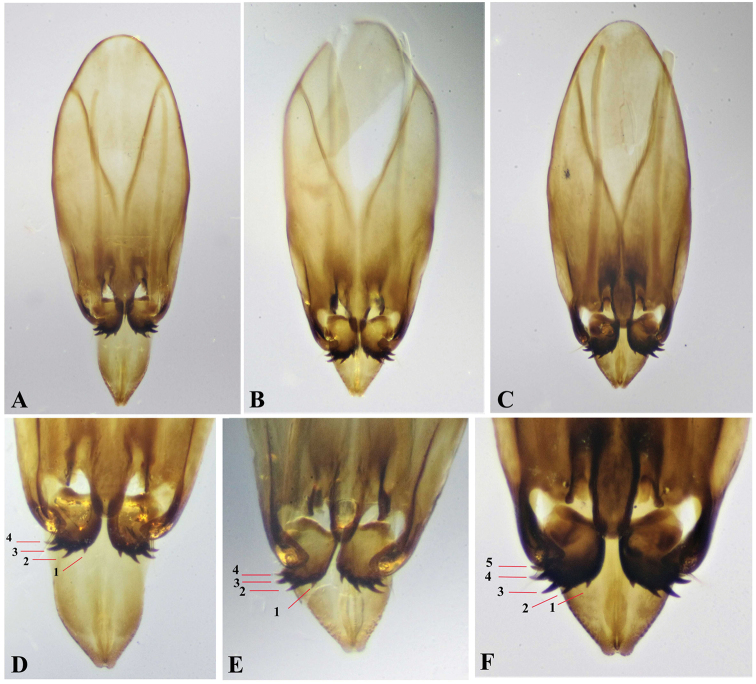
Dorsal aspect and tip of male genitalia, Paratype **A, D***Oodera omanensis* sp. nov. **B, E***Oodera rapuzzii* sp. nov. **C, F***Oodera similis* sp. nov. (numbers 1−5 indicate number of teeth on volsellae).

Similar to female. **Genitalia** (Fig. 11A, D): narrowly rounded above; volsella with four outwardly curved, sharp teeth.

#### Host record.

Anthaxia (Haplanthaxia) abdita Bílý, 1982, A. (H.) kneuckeri zabranskyi Bílý, 1995 (Buprestidae).

#### Distribution.

Oman (Dhofar governorate).

#### Remarks.

The new species, *O. omanensis*, closely resembles *O. circularicollis* Werner & Peters, *O. formosa* (Giraud), and *O. niehuisorum* Werner & Peters, but differs from them in the following:

***O. omanensis* vs. *O. circularicollis* (Morocco).** Eye large, 0.70−0.75× as high as head (small, 0.54–0.56× as high as head, in *circularicollis*); POL 1.4× OOL (as long as OOL in *circularicollis*); pronotum as long as wide, with anterior margin (collar) truncate (0.91× as long as wide, with collar virtually round in *circularicollis*); mesoscutellum almost completely lineate, finely areolate slightly before frenal line (lineate in anterior two-thirds, rugulose in posterior third in *circularicollis*); marginal vein 4× as long as stigmal vein (2.5– 3.53× as long as stigmal vein in *circularicollis*).

***O. omanensis* vs. *O. formosa* (Southern and Central Europe, Russia, Eastern United States, Eastern Canada).** Head and mesosoma blue and purplish (dark green and coppery in *formosa*); scape of antenna red-brown, with black tint apically (yellow, darkening apically in *formosa*); head width 4.5× eye distance (3.00–3.78× eye distance in *formosa*); eye 0.75× as height as head (0.55−0.68× as height as head in *formosa*); corona with structure interrupted (with structure continuous in *formosa*); pronotum with broadest part behind midlength (with broadest part at midlength in *formosa*); mesoscutellum almost completely lineate, finely areolate slightly before frenal line (lineate in anterior half to anterior two-thirds, rugulose in posterior half or third in *formosa*); profemur robust to medium, 1.92–2.00× as long as wide (usually medium to elongated, 1.94–2.33× as long as wide, in *formosa*).

***O. omanensis* vs. *O. niehuisorum* (Egypt and Israel).** Forewing partly slightly infumate (hyaline in *niehuisorum*); corona thick, 3.33−3.50× as long as wide, with structure interrupted (usually medium, 3.8–6.0× as long as wide, with structure continuous in *niehuisorum*); pronotum with broadest part behind midlength (broadest part before midlength in *niehuisorum*); mesoscutellum almost completely lineate, finely areolate slightly before frenal line (densely lineate in anterior half to anterior two-thirds, areolate in posterior half or third in *niehuisorum*); marginal vein medium, 0.92−1.00× as long as postmarginal vein (short, 0.78–0.89× as long as postmarginal vein in *niehuisorum*).

*O. omanensis* sp. nov. also resembles the new species *O. similis*, but differs from it in the following combination of characters: forewing partly infumate, with dark brown to black veins (hyaline in *O. similis*, with pale brown veins); head with bluish to purplish luster (green and coppery in *O. similis*); mesoscutellum with green basal half, violet posteriorly (entirely green in *O. similis*); pronotum distinctly narrow anteriorly, with dense lanceolate whitish setae longitudinally along the middle area, with broadest part behind midlength (distinctly broad anteriorly, with fewer setae along the middle area longitudinally, with broadest part before midlength in *O. similis*); propodeum medium, ppd.l/msc.l 0.13–15 (large, ppd.l/msc.l 0.17−0.21, in *O. similis*); volsella of male genitalia with four sharp teeth, aedeagus with parallel outer sides (with five teeth, aedeagus with strongly convex outer margins in *O. similis*).

#### Etymology.

Named in reference to the country of Oman, where the type specimen was collected.

### Oodora rapuzzii

Taxon classificationAnimaliaHymenopteraPteromalidae

Soliman & Gadallah sp. nov.

0B742A47564A5C058A279839D699B352

http://zoobank.org/BAC4F734-2978-458C-A25D-D69975B2BC5D

Figs 2C , 3C , 4C , 5 (C, G), 6C, 7C, 8C, 9(C, G), 10C, 11(B, E)

#### Material examined.

**Holotype** ♀: SAUDI ARABIA, Al-Baha (Wadi Tourabah), 12.iv.2016, leg. P. Rapuzzi, e.l. *Acacia* [KSMA]. **Paratypes**: 3♀ & 2♂: SAUDI ARABIA, Al-Baha (Al-Mikhwah, The Ain Village), 13.iv.2016, leg. D. Baiocchi, e.l. *Acacia* sp. [KSMA]; 3♂: SAUDI ARABIA, Al-Baha (Wadi Shoqab), 12.iv.2016, leg. D. Baiocchi, e.l. *Acacia* sp. [KSMA]; 1♂: SAUDI ARABIA, Al-Baha (Wadi Yabah), 15.iv.2016, leg. D. Baiocchi, e.l. *Acacia* sp. [KSMA]; 1♂: SAUDI ARABIA, Asir (Wadi Sabian, 28 km S. Muhayil), 5.iv.2017, leg. D. Baiocchi, e.l. *Acacia* sp. [KSMA]; 2♀ & 2♂: OMAN, Dhofar (Rawiyya), 16.i.2018, leg. D. Baiocchi, e.l. *Acacia* sp. [KSMA]; 1♂: OMAN, Dhofar (Mirbat), 15−18.i.2018, leg. D. Baiocchi, e.l. *Acacia* sp. [KSMA].

#### Diagnosis.

Both sexes (*N* = 16). See Table 1.

#### Description.

Female (holotype): Body length 5.6mm (excluding the ovipositor).

***Colour.*** Head black on corona, scrobal depression and occiput, coppery on face (lower face with green luster), blue on gena (Figs 5C, G, 6C); scape red-brown on basal two-thirds, rest of antenna, maxillary and labial palpi dark brown to black (Figs 3C, 5C, 7C). Mesosomal dorsum black with blue luster on pronotal disc, green on anterior half of mesoscutum, green and purple on anterior part of axilla and on all mesoscutellum, blue and purple on propodeum (Figs 8C, 9G); mesosomal venter with strong blue, green and purple luster (Fig. 4C); coxae black with strong purple luster on ventral side of procoxa (Fig. 4C); profemur black with green tint on outer face (Fig. 3C); trochaters, meso- and metafemora brown, tarsi yellow-brown (Fig. 4C). Metasoma black, with patches of green-purple on lateral sides of Gt2−4 (Figs 2C, 3C, 4C). Wings hyaline, veins brown (Fig. 10C).

***Head.*** 1.66× as wide as long (Fig. 5C); hea.w 4.56× eye.d (Fig. 5C); face setiferous foveate-reticulate, setae lanceolate, white and short (Fig. 5C); msp.l 0.43× head height (Fig. 5G); corona 0.6× as long as eye.h (Fig. 5C); POL 1.33× OOL (Fig. 6C); scape 3× as long as pedicel; clava 0.15× as long as funicle; flagellum 1.45× as long as head width; F1 0.68× as long as F2; F2 hardly longer than F3, 1.05×.

***Mesosoma.*** Pronotum pentagonal, 0.5× as long as mesonotum (Fig. 8C); mesonotum 1.5× as long as mesoscutum (Fig. 8C); mesoscutum as long as wide (Fig. 8C); mesoscutellum 0.44× as long as mesoscutum (Fig. 8C); propodeum laterally with a smooth ridge in front of the setose area (Fig. 9G); profemur 1.29× as long as protibia; pronotal disc and posterior part of mesoscutum with stout short white setae.

***Forewing*** (Fig. 10C). Forewing 2.84× as long as wide; costal cell 0.35× as long as forewing; marginal vein 0.21× as long as forewing; marginal vein 3.62× as long as stigmal vein; postmarginal vein 3.79× as long as stigmal vein.

***Metasoma*** (Fig. 2C, 3C, 4C). mts.l/mts.w = 2.45.

#### Male.

Similar to female but slightly darker in colour. **Genitalia** (Fig. 11B, E) widely rounded above; volsella with four outwardly directed, sharp teeth, the innermost one is very short compared with the others.

#### Host record.

Anthaxia (Haplanthaxia) abdita Bílý, 1982, A. (H.) cf. angustipennis (Klug, 1829), A. (H.) kneuckeri zabranskyi Bílý, 1995, A. (H.) marginifera dhofarica Bílý, 2003, A. (H.) wittmeri Bílý, 1979; *Chalcogenia halperini arabica* Bílý, 2008 (Buprestidae).

#### Distribution.

Oman (Dhofar governorate); Saudi Arabia (Al-Baha and Asir regions).

#### Remarks.

The new species closely resembles *O. formosa* (Giraud), but differs from it in the following combination of characters: wing hyaline (partly infumate in *O. formosa*); corona thick, 3.25−4.00× as long as wide, with structure interrupted (thick to medium, 3.20−6.67, structure continuous in *O. formosa*); metasomal length short to medium, 0.44−0.47× as long as body (short to long, 0.43−0.55 in *O. formosa*); ovipositor length rather long, 0.16−0.19× as long as metasoma (usually short, 0.09−0.17 in *O. formosa*); head and mesosoma with strong blue colour in some parts (never with blue evident in *O. formosa*).

#### Etymology.

This species is named in honour of Pierpaolo Rapuzzi, who participated in the breeding of this species from the dead wood of *Acacia* sp.

### Oodera similis

Taxon classificationAnimaliaHymenopteraPteromalidae

Gadallah & Soliman sp. nov.

500FC7EB1BD95539B4B55DBAEF197662

http://zoobank.org/95C405C2-217D-430F-8C70-5D27412D49D8

Figs 2D , 3D , 4D , 5 (D, H), 6D, 7D, 8D, 9(D, H), 10D, 11(C, F)

#### Material examined.

**Holotype** ♀: SAUDI ARABIA, Riyadh (Wadi Al Hesiyah, 40 km NW Riyadh), 30.iv.2017, leg. D. Baiocchi, e.l. *Acacia* sp. [KSMA]; **Paratypes**: 1♀ & 1♂, OMAN Al-Dakhiliyah (Al-Hamra), 21.i.2018, leg. D. Baiocchi, e.l. *Acacia* sp. [KSMA].

#### Diagnosis.

Both sexes (*N* = 3). See Table 1.

#### Description.

Female (holotype): Body length 6.3 mm (excluding the ovipositor).

***Colour***. Head black with slight blue-green tint on corona, scrobal depression and occiput (Figs 5D, 6D), become coppery with green luster on face, and blue on gena (Figs 5D, H); scape red-brown, rest of antenna, maxillary and labial palpi dark brown to black (Figs 3D, 7D). Mesosomal dorsum black with green and purple luster on pronotum, anterior third of mesoscutal median lobe, anterior part of axilla and on propodeum (Figs 8D, 9D, H); mesoscutellum metallic green, with slight coppery luster anteriorly (Fig. 9H); mesosomal venter blue-green, with purple luster on mesopleuron (Fig. 4D); coxae black with strong green luster on ventral side, mesocoxa mostly black (Fig. 4D); trochanters, tibiae and tarsi red-brown, protrochanter darker (Figs 3D, 4D); profemur black with purple-green tint on outer side (Fig. 3D). Metasoma black, tergites with patches of blue laterally (Fig. 2D, 3D, 4D). Wings hyaline, veins brown (Fig. 10D).

***Head.*** 1.7× as wide as long, hea.w 4.2× eye.d (Fig. 5D); face setiferous foveate-reticulate, setae lanceolate, white and short (Fig. 5D); msp.l 0.4× head height (Fig. 5H); corona 0.5× as long as eye.h (Fig. 5D); POL 1.7× OOL (Fig. 6D); scape 3.5× as long as pedicel; clava 0.13× as long as funicle; flagellum 1.3× as long as hea.w; F1 0.75× as long as F2; F2 hardly longer than F3, 1.05×.

***Mesosoma.*** Pronotum pentagonal, 0.5× as long as mesonotum (Fig. 8D); mesonotum 1.5× as long as mesoscutum (Fig. 8D); mesoscutum 0.9× as long as wide (Fig. 8D); mesoscutellum 0.5× as long as mesoscutum (Fig. 8D); propodeum with costate ridge in front of the setose area (Fig. 9H); profemur 1.3× as long as protibia.

***Forewing*** (Fig. 10D). Forewing 2.75× as long as wide; costal cell 0.4× as long as forewing; marginal vein 0.2× as long as forewing; marginal vein 3.5× as long as stigmal vein; postmarginal vein 3.66× as long as stigmal vein.

***Metasoma*** (Fig. 2D, 3D, 4D). mts.l/mts.w = 2.12.

#### Male.

Similar to female except for second and third metasomal sternites with blue reflection. **Genitalia** (Fig. 11C, F). Narrowly rounded above; volsella with five sharp, outwardly curved teeth, of which the innermost is minute.

#### Host record.

Anthaxia (Haplanthaxia) abdita Bílý, 1982, A. (H.) kneuckeri zabranskyi Bílý, 1995 (Buprestidae).

#### Distribution.

Oman (Al-Dakhiliyah governorate); Saudi Arabia (Riyadh region).

#### Remarks.

This species resembles the Oriental species *O. srilankiensis* Werner & Peters 2018 (Sri Lanka) in having the body robust; antennal scape red-brown, rest of antenna black; flagellum about 1.3× as long as head width; pronotum about 0.5× as long as mesonotum; pronotum pentagonal, with broadest part before midlength; propodeum large; forewing hyaline; marginal vein medium; ovipositor distinctly shorter than metasoma (less than 0.25× metasomal length). However, it differs from *O. srilankiensis* in the following combination of characters: body size larger, 6.30−6.5 mm in length (4.00−5.75 mm in *srilankiensis*); head and mesosoma black with metallic green, blue, purple and coppery in different parts (dark blue to blue-green in *srilankiensis*); head 1.70× as wide as long (1.28−1.48× in *srilankiensis*); head width 4.20× eye distance (3.44−3.85× in *srilankiensis*); corona 3.10−3.40× as long as wide, with structure interrupted (3.70−4.75× as long as wide, with structure continuous in *srilankiensis*); mesoscutellum almost entirely lineate, finely areolate slightly before frenal line (meoscutellum lineate in anterior third to half, rugulose in posterior half or two-thirds in *srilankiensis*); profemur robust, 1.95−2.00× as long as wide (usually medium to elongated, 1.98–2.33× as long as wide, in *srilankiensis*).

The new species resembles also the Palaearctic species, *O. niehuisorum* Werner & Peters, 2018 in having the small body size; wings hyaline; eye large; metasoma short; pronotum pentagonal, with broadest part before midlength. However, it differs from *O. niehuisorum* in the following combination of characters: head with some blue (never with blue in *niehuisorum*); antenna with scape red-brown, rest dark brown to black (scape and pedicel (except apex of pedicel) yellow, rest dark brown to black in *niehuisorum*); corona thick, 3.10−3.40× as long as wide, with structure interrupted (medium, 3.80−6.00× as long as wide, with structure continuous); mesoscutellum normal, sct.l/sct.w 0.60−0.72 (normal to slender in *niehuisorum*, 0.55−0.85); propodeum large, ppd. l/msc.l 0.17−0.21 (medium to large in *niehuisorum*, 0.12−2.15); mesoscutellum completely lineate, slightly finely areolate before frenal line (densely lineate in anterior half to anterior two-thirds, and areolate on posterior half or third in *niehuisorum*); profemur robust, fm1.l/fm1.w 1.95−2.00 (robust to medium in *niehuisorum*, 1.82−2.15); marginal vein medium, mav.l/pmv.l 1.95−1.00 (short in *niehuisorum*, 0.78−0.89); ovipositor short, ovp.l/mts.l 0.13−0.14 (short to rather long in *niehuisorum*, 0.14−0.18).

#### Etymology.

From the Latin, refers to the similarity of this species with *O. srilankiensis* Werner & Peters.

## Discussion

In the present study, four new species of the genus *Oodera* reared from dead *Acacia* trees are collected from different regions of Oman and Saudi Arabia (new locality record), with the help of beetle specialists. The study is based on morphological data of 25 specimens (13 females and 12 males). An illustrated key to Arabian species and detailed description of the new species are provided, in addition to analysis with similar valid species. Intraspecific variation is found to be slight as the number of the collected specimens is relatively small because of the rarity of this genus.

The current study is the second contribution to the study of this beautiful and interesting group of Chalcidoidea, covering a new area (Arabia) that was not considered in previous studies (example Werner and Peters 2018). Four new species are added to the world fauna thus increasing the total number to 24 species.

The world species of *Oodera* was first revised by Werner and Peters (2018), who recognized 20 valid species from which 10 are described as new species. Full descriptions of the new species, and re-description of formerly known ones are given, together with an illustrated key to world species. Few taxonomic changes are also discussed.

The main observation emerging from our study, is the strong correlation of *Oodera* fauna with the intermediate biogeographical situation of the study area. Almost, all of the studied specimens were collected from southwestern and southeastern parts of Arabia, that are exclusively Afrotropical (Larsen 1984; Burckhardt and Mifsud 1998), only one specimen is collected from Riyadh (Palaearctic). A hypothesis that is supported by Werner and Peters (2018) who concluded that *Oodera* species seemingly prefer warmer to temperate regions.

However, because of the biodiversity richness of Arabia, due to its rich floristic diversity, more species of this genus are expected to occur. Therefore, further collections and studies are still needed to clarify the distribution of this genus in other parts of this area.

## Supplementary Material

XML Treatment for Oodera

XML Treatment for Oodera arabica

XML Treatment for Oodera omanensis

XML Treatment for Oodora rapuzzii

XML Treatment for Oodera similis
